# VEXAS without vacuoles: Linking genotype to phenotype

**DOI:** 10.1002/jha2.1016

**Published:** 2024-10-05

**Authors:** Sara Zhukovsky, Anton Rets, Tawnie Braaten, Ami B. Patel

**Affiliations:** ^1^ Department of Internal Medicine University of Utah Salt Lake City Utah USA; ^2^ Department of Pathology University of Utah Salt Lake City Utah USA; ^3^ Division of Rheumatology University of Utah Salt Lake City Utah USA; ^4^ Division of Hematology and Hematologic Malignancies Huntsman Cancer Institute University of Utah Salt Lake City Utah USA

**Keywords:** bone marrow pathology, cancer genetics, inflammation, MDS

## Abstract

**Introduction:**

VEXAS syndrome is a rare condition characterized by somatic mutations in the ubiquitin‐like modifier activating enzyme 1 (*UBA1*) gene and a constellation of clinical/morphologic findings, including the presence of cytoplasmic vacuoles within bone marrow hematopoietic cells.

**Methods and objectives:**

In this report, we present a case of a male patient diagnosed with VEXAS‐associated myelodysplastic syndrome following the detection of a non‐canonical *UBA1* p.Gly477Ala variant whose bone marrow biopsy revealed a conspicuous absence of cytoplasmic vacuolization in hematopoietic cells. This case prompts a comprehensive review of the existing literature on the significance and pathobiology of vacuolization in the context of VEXAS and *UBA1* mutations.

## 1 INTRODUCTION

VEXAS (vacuoles, E1 enzyme, X‐linked, autoinflammatory, somatic) syndrome is a rare clinical entity first described in 2020 and characterized by acquired mutations typically involving p. Met41 in the ubiquitin‐like modifier activating enzyme 1 (*UBA1*) gene [1]. It is almost exclusively found in men, though rare cases have been described in women with acquired or inherited monosomy X [2]. While the median age of diagnosis is 61 years, the syndrome has been reported in patients as young as 29 years [3]. VEXAS presents as an adult autoinflammatory disease with profound constitutional symptoms including fevers, night sweats, weight loss, and an array of rheumatologic manifestations such as myalgias, arthralgias, chondritis, vasculitis, and hematologic abnormalities including cytopenias and paraproteinemia. There is a well‐established association between VEXAS and the development of hematologic malignancies, with an estimated 30%–50% of patients progressing to myelodysplastic syndrome (MDS) [1, 4–8], though recent evidence suggests this may be an overestimate [9]. Diagnosis of VEXAS requires the detection of a somatic mutation within the *UBA1* gene encoding the E1 enzyme that is responsible for catalyzing the first step of ubiquitylation‐dependent intracellular protein degradation [1]. Another hallmark feature of VEXAS is the presence of cytoplasmic vacuoles within bone marrow hematopoietic cells, which occurs in over 90% of VEXAS cases [1, 4–8] and has been observed in promyelocytes, myelocytes, erythroid precursors, and blasts [10]. In this report, we present the case of a male patient diagnosed with VEXAS‐associated MDS following the detection of a non‐canonical *UBA1* p.Gly477Ala variant whose bone marrow biopsy revealed a conspicuous absence of cytoplasmic vacuolization in hematopoietic cells. This case prompts a comprehensive review of the existing literature on the significance and pathobiology of vacuolization in the context of VEXAS and *UBA1* mutations.

### 1.1 Patient case

A 72‐year‐old man with a past medical history of gout, coronary artery disease, thyroid cancer in remission, and obstructive sleep apnea presented with inflammatory polyarthritis in 2016. His symptoms included joint pain and intermittent swelling in his bilateral feet, elbows, wrists, hands, and right knee. Initially, this was thought to be polyarticular gout but joint aspiration demonstrated a lack of crystals and the patient failed to improve with aggressive anti‐gout therapies. He was referred to Rheumatology, but serologic workup, including testing for anti‐cyclic citrullinated peptide, rheumatoid factor, anti‐neutrophilic cytoplasmic antibodies, ANA, double‐stranded DNA, Ro/SSA, La/SSB, Smith, and anti‐mitochondrial antibodies were negative. The patient was subsequently diagnosed with seronegative rheumatoid arthritis. For several years his course was complicated by multiple treatment failures including methotrexate, tocilizumab, golimumab, adalimumab, and infliximab. In 2021, while on infliximab 5 mg/kg he was found to have ileal inflammation on screening colonoscopy, with pathology showing active ileitis and crypt architectural distortion. The patient did not have any gastrointestinal symptoms at that time. He underwent a gastroenterology consultation and was diagnosed with Crohn's disease. His most recent colonoscopy showed chronic inactive ileitis while on infliximab 10 mg/kg.

In 2017 the patient was found to have new thrombocytopenia and neutropenia in the background of chronic anemia (baseline hemoglobin of 10 g/dL). He was also found to have mild splenomegaly (spleen size 14.5 cm). Initial bone marrow biopsy showed hypercellular bone marrow (60%) with trilineage hematopoiesis, erythroid hyperplasia (M:E ratio 0.3), increase in storage iron with rare ring sideroblasts and hypogranular neutrophils. Peripheral blood hemolysis workup was unremarkable. Cytogenetics showed normal male karyotype. His methotrexate and tocilizumab were held with the assumption that his pancytopenia was medication‐induced. Following a lack of improvement in his counts, the patient was referred to Hematology and underwent a repeat bone marrow biopsy in 2019 showing hypercellular marrow (80%–90%) with trilineage hematopoiesis, erythroid hyperplasia, mild dyserythropoiesis and mild dysmegakaryopoiesis. Cytogenetics and MDS fluorescent in‐situ hybridization panel were normal and the myeloid panel by massive parallel sequencing (next‐generation sequencing) showed a variant of unknown significance in the *NSD1* gene at VAF 48.3%.

**FIGURE 1:**
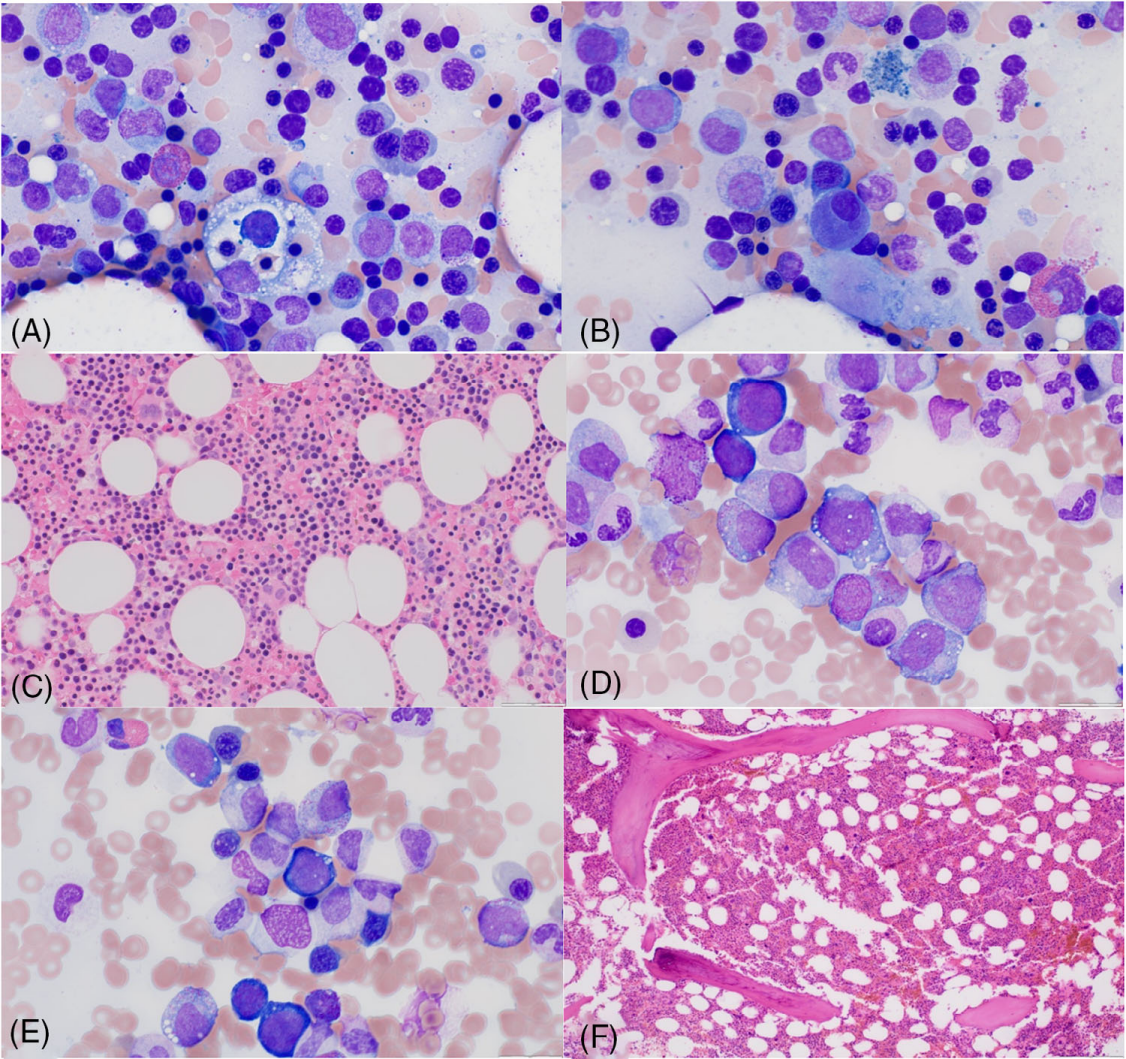
(A, B) Aspirate smear (modified Giemsa stain) from this patient (UBA1Gly477Ala) with hemophagocytes and dysplastic megakaryocyte (micromegakaryocyte) without cytoplasmic vacuolization. Image1C: Core biopsy (hematoxylin and eosin‐stained) from this patient (UBA1Gly477Ala) with well‐formed erythroid islands and dysplastic megakaryocytes. Image (1D–F) Vacuolization in erythroid precursors (1D), myeloid precursors (1E), and core biopsy with hypercellular marrow with myeloid and megakaryocyte hyperplasia and atypical megakaryocytes (1F) in a patient with UBA1Met41Leu.

**FIGURE 2:**
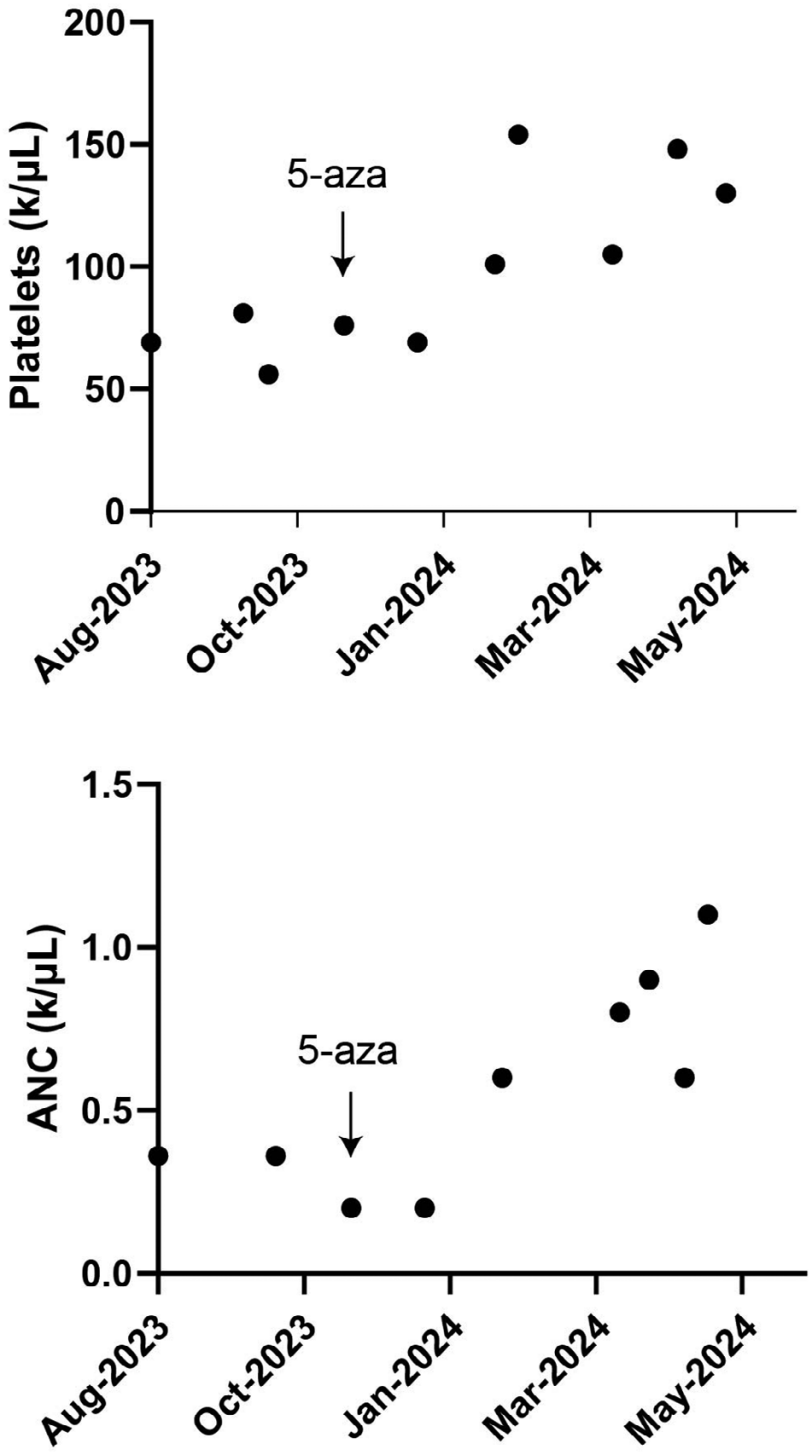
Improvement in the patient's platelet and absolute neutrophil count (ANC) following initiation of 5‐azacitidine.

The patient was closely monitored and blood counts waxed and waned for several years until his neutrophil count down‐trended to a nadir of 0.2 × 10^9^/L. Repeat bone marrow biopsy in 2023 revealed normocellular marrow (50%) with erythroid‐predominant trilineage hematopoiesis and dysmegakaryopoiesis with 1% blasts by aspirate counts that were immunophenotypically atypical by flow cytometry. M:E ratio was again low at 0.3. Repeat myeloid sequencing panel showed an atypical *UBA1* p.Gly477Ala mutation with a variant allele frequency of 81.5% confirming the diagnosis of VEXAS. Interestingly, no cytoplasmic vacuoles were observed in the patient's bone marrow (Figure 1). Of interest, occasional hemophagocytes were appreciated in the marrow. Due to a significant morphologic overlap between VEXAS and MDS (cytopenias, hypercellular marrow with morphologic atypia) as well as our limited understanding of the relationship between the two, a diagnosis of MDS in VEXAS patients may be very challenging. In our case, persistent pancytopenia, classic dysplastic morphology of megakaryocytes (small, hypolobate forms), and immunophenotypic atypia of myeloid blasts along with the abovementioned findings raised a strong suspicion of at least emerging MDS. The patient was started on 5‐azacitidine with modest improvement in his neutrophil and platelet counts (Figure 2), but slight worsening of baseline anemia, suggesting that certain cytopenias in VEXAS may be less responsive to hypomethylating agent therapy, such as those suspected to be related to chronic inflammation, which may in turn improve with dedicated anti‐inflammatory treatment. Ultimately, our patient was referred to the National Institutes of Health for consultation and consideration of an allogeneic bone marrow transplant.

## 2 DISCUSSION

The presence of cytoplasmic vacuoles in bone marrow precursors is a sensitive but non‐specific marker for VEXAS [11]. Cytoplasmic vacuolization in myeloid and erythroid progenitors has been described in various conditions such as copper and zinc deficiency, acute myeloid leukemia, and alcohol toxicity [12]. Toxic vacuolization in circulating neutrophils represents a common response to infection and inflammation indicative of enhanced phagocytosis [13]. Although vacuolization of hematopoietic progenitor cells is a hallmark feature in VEXAS, the significance of vacuolization or lack thereof in this disease remains ill‐defined. Our patient had an indolent disease course, with symptom onset nearly 7 years prior to MDS diagnosis and a notable lack of typical inflammatory features such as fevers. A previously reported VEXAS patient with *UBA1* p.Gly477Ala was also initially devoid of bone marrow vacuolization two years into the onset of inflammatory symptoms but developed this feature later in his disease course [6]. Another unusual feature observed in our case was the low M:E ratio and erythroid hyperplasia noted on the patient's bone marrow biopsies in the absence of peripheral blood hemolysis. This differs from bone marrow features typically found in VEXAS patients harboring Met41 substitutions, in which high M:E ratios and erythroid hypoplasia are classic findings, and further suggests that non‐canonical mutations may confer distinct histopathological and possibly, clinical, phenotypes [4, 14].

In Table 1, we present the summary of the clinical characteristics associated with canonical Met41 and rare non‐Met41 VEXAS‐causing *UBA1* mutations across several case series. The absence of bone marrow progenitor vacuoles was observed in 5/57 reviewed VEXAS cases, including those harboring *UBA1* variants p.Gly477Ala, p.Ala478Ser, p.Asp506Gly, and two splice site mutations, (*c.118‐9_118‐2del*). All VEXAS cases with Met41 mutations reported vacuolization while lack of vacuolization was exclusively present in non‐Met41 mutations. Clinical features of VEXAS such as fevers, skin involvement, macrocytic anemia, and arthralgias were prevalent with all pathogenic *UBA1* variants, regardless of vacuolization status. Concurrent MDS was noted in all cases lacking vacuolization (5/5). While data pertaining to the timing of disease progression was infrequently reported, cases without vacuolization had a minimum of 2 years from symptom onset to progression to MDS (*n* = 2), suggestive of a more indolent disease trajectory.

**TABLE 1:** Review of clinical features of reported Met41 and non‐Met41 ubiquitin‐like modifier activating enzyme 1 (*UBA1*) variants.

*UBA1* variant	Bone marrow vacuoles	MDS	Fever	Skin involvement	Pulmonary infiltrate	Arthritis/Arthralgia	Macrocytic anemia	Years of auto‐inflammatory symptoms prior to MDS diagnosis	Reference
p.Met41Thr (*n* = 25) p.Met41Val (*n* = 11) p.Met41Leu (*n* = 6)	42/42	15/42	39/42	38/42	24/42	9/9*	38/42	NA	Poulter et al., Beck et al, Bourbon et al.
p.Ser56Phe (*n* = 1)	NA	1/1	1/1	1/1	0/1	1/1	1/1	NA	Poulter et al.
Splice mutations: p. (splice) (c.118‐1G > C) (*n* = 1) splice motif at the junction of intron 3 and exon 3 (*n* = 2) c.118‐9_118‐2del (*n* = 2)	3/5 (No vacuolization in 2/2 c.118‐9_118‐2del mutations)	4/5	3/4* (NA in 2/2 c.118‐9_118‐2del mutations)	5/5	1/5	1/4* (NA in 2/2 c.118‐9_118‐2del mutations)	4/5	1/5: 3 years (c.118‐9_118‐2del mutation) 4/5: NA	Poulter et al., Bourbon et al.
p.Ala478Ser (*n* = 3)	1/2*	3/3	1/2*	2/2*	0/2*	1/2*	1/2*	NA	Collins et al., Sakuma et al.
p.Asp506Asn (*n* = 1)	NA	NA	1/1	1/1	0/1	NA	1/1	NA	Collins et al.
p.Asp506Gly (*n* = 1)	0/1	1/1	1/1	0/1	0/1	0/1	1/1	NA	Collins et al.
p.Tyr55His (*n* = 1)	1/1	1/1	1/1	1/1	0/1	NA	1/1	NA	Collins et al.
p.Gly477Ala (*n* = 2)	1/2	2/2	1/2	2/2	0/2	2/2	2/2	Patient 1: 4 years Patient 2: 7 years (no vacuoles)	Stiburkova et al.
Concurrent mutations: p.Ile894Ser and p.Asn606Ile (*n* = 1)	1/1	1/1	1/1	1/1	0/1	0/1	0/1	Several Months	Sakuma et al.
Concurrent mutations: p.Tyr55His, p.Ile894Phe (*n* = 1)	1/1	1/1	NA	NA	NA	NA	NA	NA	Sakuma et al.
p.Arg182His	1/1	1/1	NA	NA	NA	NA	NA	NA	Sakuma et al.
p.Glu597Ala	NA	0/1	NA	NA	NA	NA	NA	NA	Sakuma et al.
p.Ser621Cys	1/1	1/1	NA	NA	NA	NA	NA	NA	Sakuma et al.
p.Pro749Leu	NA	1/1	NA	NA	NA	NA	NA	NA	Sakuma et al.
p.Pro1014Leu/canonical splice site	NA	1/1	NA	NA	NA	NA	NA	NA	Sakuma et al.

* Represented as a fraction of evaluable cases only.

VEXAS was initially described following a genotype‐first screening approach of patients with undiagnosed inflammatory disorders. This study identified acquired somatic missense mutations at p. Met41, encoding the start codon in exon 3 of *UBA1*, in 25 male patients [1]. More than 70% of published VEXAS cases have a p.Met41 substitution resulting in loss of exon 3 in the cytoplasmic isoform of UBA1 (UBA1b), resulting in a hypomorphic enzyme (UBA1c) with reduced catalytic activity that leads to decreased cellular polyubiquitination [12]. Splice site mutations directly upstream of exon 3 have been described in 6% of VEXAS patients and are also believed to lead to exon 3 deletion [12]. Reduced cytoplasmic UBA1 ubiquitination is thought to cause accumulation of misfolded proteins and endoplasmic reticulum stress, resulting in activation of the unfolded protein response (UPR) and inciting the inflammatory pathogenesis of the disease [1]. Recently, non‐canonical somatic UBA1 mutations at non‐Met41 and non‐exon 3 splice sites have been further characterized [4, 6–8]. Collins et al. reported that non‐canonical VEXAS‐causing *UBA1* mutations located close to the ubiquitin‐ and ATP‐binding sites in the adenylation pocket exhibit reductions in ubiquitin adenylation and thioester formation and promote aberrant oxyester production, ultimately impairing ubiquitin transfer to cytoplasmic E2 enzymes and inactivating both nuclear and cytoplasmic UBA1 catalytic activity. In particular, the UBA1 p.Gly477Ala variant observed in our patient was found to be 2‐fold more deficient in ubiquitin adenylation and thiolation and displayed significant defects in transthiolation as compared to wild‐type UBA1 [8].

Based upon our patient's case and review of the literature, we speculate that non‐canonical *UBA1* mutations causing E2 dysregulation may result in less severe inflammatory VEXAS phenotypes than Met41 mutations leading to loss of UBA1b and production of UBA1c. As such, the presence or absence and extent of bone marrow vacuolization may serve as a surrogate measure for the degree of cellular stress and inflammation related to the UPR. The presence of lipid droplets and cellular debris, such as disintegrating mitochondria, in vacuoles from VEXAS patients has led to the thought that they may in fact be autophagosomes [15], which is consistent with the essential role of the UPR in autophagy regulation [16, 17].

An enhanced understanding of the nature and significance of hematopoietic cell vacuolization in VEXAS may help identify cases characterized by high levels of inflammation and more severe disease trajectories, potentially informing prognostic risk models and treatment paradigms. Additional studies correlating vacuolization with inflammatory marker levels, clinicopathological disease features, and specific *UBA1* variants are needed and will be complemented by mechanistic insights from preclinical models.

## AUTHOR CONTRIBUTIONS

Sara Zhukovsky and Ami B. Patel conceived of this project performed the literature review, and wrote the initial draft of the manuscript. Ami B. Patel, Anton Rets, and Tawnie Braaten participated in clinical care. All authors contributed to draft revisions and approved the final version of the manuscript.

## CONFLICT OF INTEREST STATEMENT

The authors declare no conflict of interest.

## ETHICS STATEMENT

The authors have confirmed ethical approval statement is not needed for this submission.

## PATIENT CONSENT STATEMENT

All patient information has been de‐identified to maintain confidentiality.

## CLINICAL TRIAL REGISTRATION

The authors have confirmed clinical trial registration is not needed for this submission.

## Data Availability

The data that support the findings of this study are available from the corresponding author upon reasonable request.
